# Sprint Interval Training and Continuous Aerobic Exercise Training Have Similar Effects on Exercise Motivation and Affective Responses to Exercise in Patients With Major Depressive Disorders: A Randomized Controlled Trial

**DOI:** 10.3389/fpsyt.2018.00694

**Published:** 2018-12-21

**Authors:** Markus Gerber, Alice Minghetti, Johannes Beck, Lukas Zahner, Lars Donath

**Affiliations:** ^1^Department of Sport, Exercise and Health, University of Basel, Basel, Switzerland; ^2^Clinic Sonnenhalde, Riehen, Switzerland; ^3^Department of Intervention Research in Exercise Training, German Sport University Cologne, Cologne, Germany

**Keywords:** affective response, exercise, fitness, major depressive disorder, motivation, physical activity, randomized controlled trial, sprint interval training

## Abstract

**Background:** Sprint interval training (SIT) has become increasingly popular and is seen as a promising exercise strategy to increase fitness in healthy people. Nevertheless, some scholars doubt the appropriateness of a SIT training protocol for largely physically inactive populations. SIT might be too arduous, and therefore contribute to feelings of incompetence, failure, and lower self-esteem, which may undermine participants' exercise motivation. Therefore, we examined whether participation in 12 SIT sessions would lead to different changes in self-determined motivation, affective responses to exercise, cardiorespiratory fitness, physical activity, and depressive symptom severity compared to aerobic exercise training (CAT) in a sample of patients with major depressive disorders (MDD).

**Methods:** Two groups of 25 patients (39 women, 11 men) with unipolar depression were randomly assigned to the SIT or CAT condition (*M* = 36.4 years, *SD* = 11.3). Data were assessed at baseline and post-intervention (three weekly 35-min sessions of SIT/CAT over a 4-week period). Self-determined exercise motivation was assessed with a 12-item self-rating questionnaire, affective valence was assessed in each session, prior, during, and after the exercise training using the Feeling Scale (FS). Cardiovascular fitness was measured with a maximal bicycle ergometer test, self-perceived fitness with a 1-item rating scale, physical activity with the International Physical Activity Questionnaire (IPAQ-SF), and depressive symptom severity with the Beck Depression Inventory II (BDi-II).

**Results:** The SIT and CAT groups did not differ with regard to their changes in self-determined motivation from baseline to post-intervention. Participants in the SIT and CAT group showed similar (positive) affective responses *during* and *after* the training sessions. Cardiorespiratory fitness, self-perceived fitness and depressive symptom severity similarly improved in the SIT and CAT group. Finally, significant increases were observed in self-reported physical activity from baseline to post-intervention. However, these increases were larger in the CAT compared to the SIT group.

**Conclusion:** From a motivational point of view, SIT seems just as suited as CAT in the treatment of patients with MDD. This is a promising finding because according to self-determination theory, it seems advantageous for patients to choose between different exercise therapy regimes, and for their preferences with regard to exercise type and intensity to be considered.

## Introduction

Major depressive disorders (MDD) are among the most common non-communicable disorders worldwide (1). Besides standard pharmaceutical and behavioral treatment of MDD (2), exercise therapy has gained increasing recognition during recent years and is now part of the WHO guidelines for the standard treatment of depression (3). In line with this, several national health foundations recommend exercise therapy as first- or second-line strategies in the treatment of MDD (4). These recommendations reflect empirical evidence that exercise therapy has the potential of improving depressive symptoms in patients with MDD (5, 6). Prior studies suggest that exercise therapy has similar effects to pharmacological treatment (7), and is associated with increased remission rates even among patients with treatment-resistant depression (8). In patients with MDD, exercise therapy also results in improved cardiorespiratory fitness (9). This is crucial as patients with psychiatric disorders have increased risk of developing cardiovascular diseases and premature cardiovascular disease-related mortality (10).

The majority of previous exercise trials in MDD focused on continuous aerobic exercise training (CAT). However, alternative forms of exercise training such as high intensity interval training (HIIT) have proven to have an even more favorable impact on participants' cardiorespiratory fitness compared to CAT in healthy populations (11–13) and patients with chronic diseases (14). Some researchers therefore argue that HIIT can be considered a time-efficient and effective alternative to CAT (15). Meanwhile, researchers have started to examine the impact of HIIT in psychiatric patients, including patients with schizophrenia and MDD (16, 17).

In the general population, sprint interval training (SIT) has become increasingly popular, and is seen as a promising exercise strategy to increase fitness in healthy, already physically active young people (18). SIT is a specific mode of HIIT, involving intermittent bouts of nearly “all out” efforts or intensities corresponding to ≥100% of power or speed associated with an individual's VO_2_max, interspersed by periods of active or passive recovery (19). Compared to CAT or continuous high-intensity training protocols, the overall caloric expenditure of SIT is considerably lower (down to 10%), but in terms of metabolic adaptations, SIT is similarly effective (20, 21). While in patients suffering from MDD supervised aerobic exercise has been applied at moderate to high intensity levels (22), little is known about the efficacy of SIT in this specific patient population.

Previously published findings from our research group suggest that similar improvements occur in cardiorespiratory fitness (VO_2_max), cardiovascular health markers (blood pressure, arterial stiffness), and depressive symptomatology in patients with MDD who participated in 12 sessions of SIT or CAT (23, 24). These promising findings notwithstanding, some scholars doubt whether HIIT or SIT are appropriate training protocols for largely physically inactive populations (25, 26). These scholars argue that proponents of HIIT/SIT have almost exclusively focused on physiological adaptation, whereas the psychological side has been neglected. Particularly, they argue that HIIT/SIT might be considered too arduous by untrained/sedentary participants [cp. (27)], and therefore contribute to feelings of incompetence, failure, and lower self-esteem, which in turn may undermine participants' motivation to engage in exercise and sport activities (28). Moreover, while it is acknowledged that HIIT/SIT might be positively perceived by some participants [e.g., recreational exercisers; cp. (29)], critics hypothesize that in the majority of physically inactive or untrained individuals, engagement in SIT will trigger negative affective responses [cp. (30, 31)]. This again may have a negative impact on participants' intrinsic motivation and their future physical activity behavior (32, 33). Following Batterham (26), however, much of this criticism is unjustified. Firstly, critics seldom take into account that different HIIT protocols may have different impacts on participants' affective responses. Secondly, findings regarding HIIT protocols are often extrapolated from high-intensity continuous exercise. Thirdly, recent evidence suggests that affective responses to HIIT are similarly positive (or better) to CAT even among inexperienced exercisers (34).

In summary, we observe a heated debate regarding the pros and cons of HIIT/SIT in sedentary populations. While we acknowledge that HIIT/SIT have great potential to trigger positive physical adaptations, we also believe that caution is required before recommending HIIT/SIT to sedentary populations or patients with psychiatric disorders as a viable training protocol. As emphasized by Hardcastle et al. (25), more research is needed to find out how such populations respond to HIIT/SIT from a psychological point of view. This claim is in line with Janney et al.'s (35) opinion that understanding patients' perspectives and preferences is essential if physical activity is to be integrated effectively in the care for psychiatric disorders. Therefore, the primary purpose of the present study was to examine whether participation in 12 sessions of SIT would lead to different changes in self-determined sport and exercise motivation (primary outcome), compared to CAT in a sample of patients with MDD. The secondary purpose was to examine whether SIT and CAT trigger different affective responses during and after exercise participation. Finally, we aimed at analyzing whether SIT and CAT resulted in similar improvements in cardiorespiratory fitness, subjectively perceived fitness, self-reported physical activity levels, and depressive symptoms. Given the exploratory nature of our research questions, no specific hypotheses were formulated.

## Methods

### Participants and Procedures

**Figure 1:**
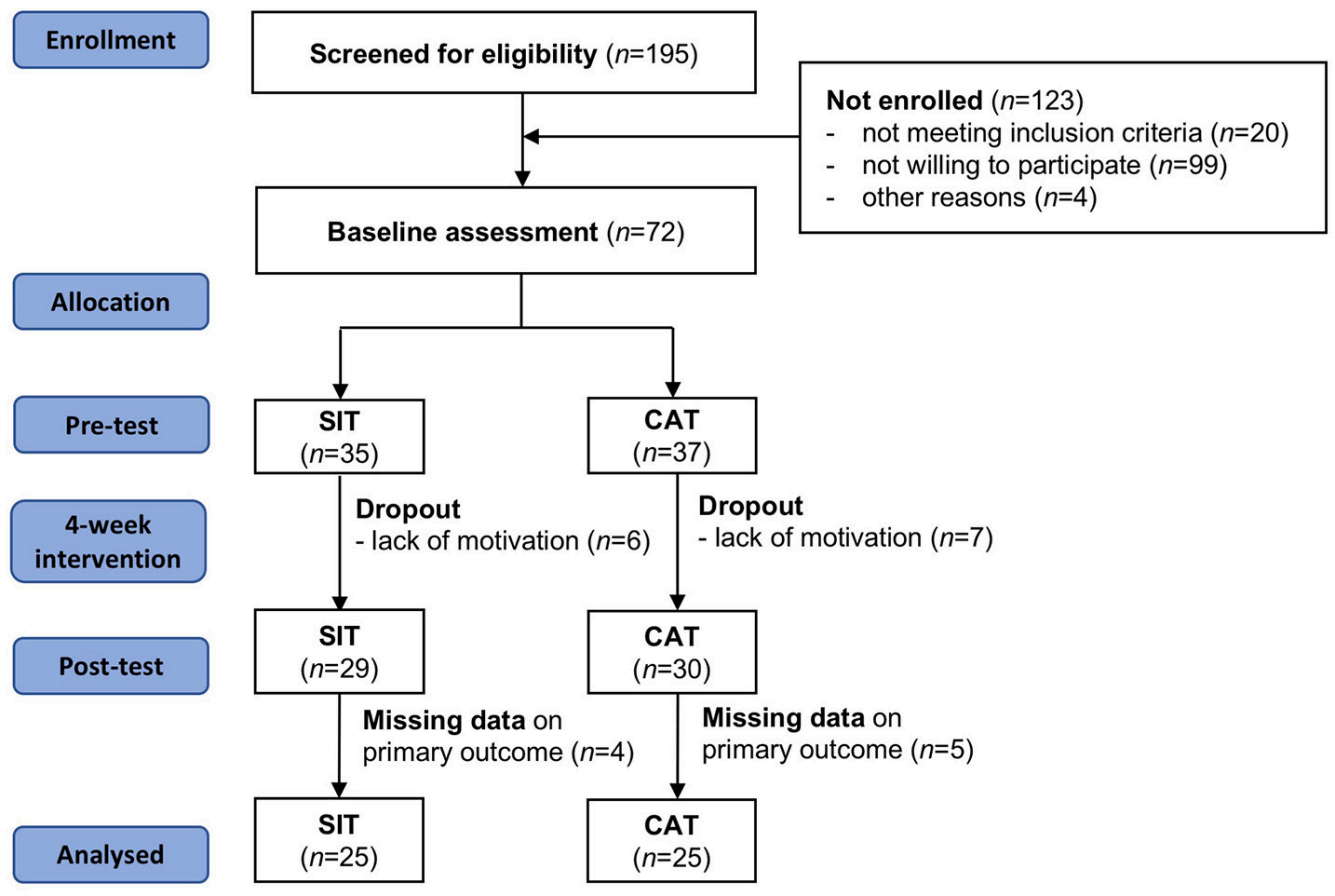
Participant flow chart.

A two-armed randomized controlled trial design was conducted to address the study questions. Eligible participants were in-patients receiving treatment at the Clinic Sonnenhalde (Riehen, Switzerland). This psychiatric clinic is located in the Northwestern, German-speaking part of Switzerland. Participants were approached and generally informed about the study by their treating psychiatrist. More detailed information about the procedures was provided by a research assistant. Prior to study enrollment, all patients provided written informed consent. As shown in Figure 1, 170 patients were screened for eligibility. Thereof, 123 participants were not enrolled in the study, mainly because they did not meet the inclusion criteria (*n* = 20) or they were not interested in participation (*n* = 99). Based on *t*-tests and χ^2^-tests, these participants did not differ from the final study population in terms of age and gender (*p* > 0.05).

The main inclusion criterion was the clinical diagnosis of a single episode of one of the following mood affective disorders, as defined by the International Statistical Classification of Diseases and Related Health Problems 10th Revision (ICD-10): F32.1: major depressive disorder, single episode, moderate. F32.2: major depressive disorder, single episode, severe without psychotic features. F33.1: major depressive disorder, recurrent, moderate. F33.2: major depressive disorder, recurrent severe without psychotic feature. Patients were excluded if they had any further psychiatric diagnoses such as (a) eating disorders such as anorexia, bulimia, or binge-eating, (b) addiction disorder or current detoxification treatment, (c) schizophrenia, (d) bipolar disorder, (e) panic disorder, and/or (f) somatic disorders such as cardiovascular diseases, stroke or thrombosis, epilepsy or other neurological disorders, pulmonary diseases, diabetes, or obesity (BMI ≥ 30).

Random assignment with minimization method (www.ncbi.nlm.nih.gov/pubmed/12505244) and the strata: (baseline depression severity, gender, age, and BMI), was applied to allocate patients to the SIT or CAT condition, using an established web-based spreadsheet (http://sportscience.sportsci.org). Data were assessed in the week prior to the beginning and after completion of the intervention. In addition, affective valence was assessed before, during, and after each exercise session. The intervention period lasted 4 weeks and consisted of 3 weekly exercise sessions of either SIT or CAT. Patients received pharmacological treatment as prescribed by their physicians throughout the entire intervention period. Moreover, patients continued with their usual (multimodal) treatment (including milieu therapy, individual and group psychotherapy and creative arts therapy).

Ethical approval was obtained from the local ethical review board (Ethical Committee of Northwestern and Central Switzerland [EKNZ]; project number: 2014-374). An a-priori power calculation showed that to detect a large effect (*f* = 0.40) in repeated measures analyses of variance (rANOVAs), a minimal sample size of 52 participants was needed (α error probability = 0.05; Power = 0.80, number of groups = 2).

### Exercise Intervention

Patients assigned to the SIT or CAT condition participated in 3 weekly exercise sessions (Mondays, Wednesdays, Fridays) during a 4-week period with each session lasting 35 min (including 5 min of warming-up and 5 min of cooling-down). Each session was performed under supervision of a trained exercise coach. In order to be included in the analyses, participants had to complete 11 of the 12 training sessions. SIT was based on a Wingate-based interval protocol of 25 repetitions of 30 s of high-intensity intervals at 80% of VO_2_max on a bicycle ergometer (ErgoSelect 300, Ergoline). Each of these intervals was followed by 30 s of complete rest during which the participants remained seated on the ergometer (36). CAT consisted of 20 min of aerobic exercise training on the same ergometer device as the SIT group. The intensity was held at a constant 60% of participants' VO_2_max. Based on the American College of Sports Medicine's metabolic calculation formula for leg cycling (37), the two exercise conditions can be considered calorically equivalent. During the entire study period, no serious adverse events occurred.

### Measures

#### Exercise and Sport Motivation

The primary outcome, exercise and sport motivation, was assessed with four indices, each consisting of three items (38). In line with Deci and Ryan's (39) definition of self-determination and the Hierarchical Model of Intrinsic and Extrinsic Motivation (40), motivation was regarded as a continuum with varying degrees of self-determination. Intrinsic regulation reflects the highest level of motivation (exercise and sport are performed for their own sake, because the activity is pleasant and fun), followed by identified regulation (exercise and sport are performed because the consequences are considered valuable; e.g., because they help a person regulate current mood states or deal with stress). Whereas, these two regulation modi reflect self-concordance, introjected, and external regulation constitute two forms of non-self-determined motivation. In the case of introjected regulation, motivation for exercise and sport is intra-personally controlled, but not self-determined (the person engages in the activity, because otherwise he/she would feel guilty). Finally, exercise and sport are externally regulated if people engage in these activities because they feel forced. Answers are given on a 6-point Likert-scale with anchors from 1 (not at all true) to 6 (completely true). For each index, the mean was calculated with higher scores indicating that a particular regulation modus is more dominant. In addition, a self-determination index can be computed, using the following formula: (intrinsic motivation ^*^ 2) + (identified motivation ^*^ 1) + (introjected motivation ^*^ −1) + (extrinsic motivation ^*^ −2). Scores range from −15 to +15, with higher scores reflecting higher self-determined motivation.

#### Affective Valence

Affective valence was assessed with the single-item Feeling Scale (FS), an 11-point bipolar measure of pleasure and displeasure, with scores ranging from −5 (very bad) to +5 (very good) (41). Participants were asked to indicate “how do you feel right now?” Previous research suggests that only moderate correlations exist between the FS and perceived exertion, showing that they are related, but distinctly different constructs (41). Evidence exists that the FS is sensitive to alterations in exercise-intensity during exercise training (42), that positive valence is associated with higher enjoyment of acute exercise (43), and that a more positive affective valence during exercise is associated with more voluntary engagement in moderate-to-vigorous physical activity (MVPA) (33). In each session, the participants completed the FS three times, before the warm-up, in the middle of SIT or CAT, and after the cool-down.

#### Cardiorespiratory Fitness

Cardiorespiratory fitness was measured with a bicycle ergometer (Ergometrics 900®, Ergoline) to establish maximal heart rate (HRmax) and maximal oxygen uptake (VO_2_max). To this end, a ramp-protocol was used with regular increases of intensity of 10 W/min until subjective perceived exhaustion (10 on Borg Perceived Exertion Scale) (44), beginning on a level of 25 W. Pedaling cadence was kept constant intra-individually (60–80 revolutions per minute), taking into account subjective pedaling comfort. After having reached exhaustion, participants continued bicycling for 3 min (10 W, 30 revolutions per minute). Resting heart rate (HR; Polar RS400, Polar® Electro, Kempele, Finland) was assessed 5 min prior to the start of the bicycle protocol. Breath-by-breath spirometric gas-exchange data (Metamax 3b, Cortex, Leipzig, Germany) were recorded every 10 s via a facemask. VO_2_max was defined as the highest oxygen uptake averaged over a period of 30 s.

#### Perceived Fitness

A single item was used to assess perceived physical fitness ranging from 1 (very poor fitness) to 10 (excellent fitness) (45). Previous research has shown that this item is correlated with objective physical fitness and perceived well-being (46).

#### Self-Reported Physical Activity

Self-reported MVPA was assessed with the International Physical Activity Questionnaire—Short Form (IPAQ-SF) (47). Previous research showed that reasonable correlations exist between this instrument and accelerometer-based data (48). Referring to the past seven days, participants reported the number of days (0 to 7) during which they performed (a) vigorous physical activity (VPA; exercise or participation in high-intensity activities and sports such as aerobics or fast bicycling), and (b) moderate physical activity (MPA; low-intensity sports such as bicycling at a regular pace). Participants also indicated the average duration (in minutes) of these activities. Multiplication of frequency and duration scores resulted in an estimate of weekly hours invested in VPA and MPA. Following the recommendations of the Centers for Disease Control and Prevention (CDC), participants who reported either (a) ≥150 min of MPA per week, (b) ≥75 min of VPA per week or (c) an equivalent mix of MPA and VPA of ≥150 min per week, can be considered as sufficiently physically active (see http://www.cdc.gov/physicalactivity/everyone/guidelines/adults.html).

#### Depressive Symptom Severity

The German version (49) of the Beck Depression Inventory II (BDI-II) (50) was used to assess depressive symptoms severity. The BDI-II is composed of 21 items, assessing a range of affective, behavioral, cognitive, and somatic symptoms, which reflect unipolar depression (e.g., “I am so unhappy/sad that I can't stand it.”). Four response options are offered ranging from 0 to 3 and describing increasing levels of depressive symptomatology. Items are summed up to generate an overall index with scores ranging from 0 to 63, with higher scores reflecting more severe depressive symptoms. Evidence of the validity and reliability of the BDI-II has been reported previously (51). Scores of the BDI-II can be interpreted in the following way (52): no depression (0–9 points), mild depression (10–18 points), moderate depression (19–29 points), and severe depression (30–63 points). In the present sample, the Cronbach's alpha was 0.92 at baseline and 0.93 post-intervention.

#### Potential Confounders

The following confounders were considered: sex, age, height, body weight, BMI (kg/m^2^), smoking status, blood pressure (systolic and diastolic), and use of psychopharmacological medication.

### Statistical Analyses

Descriptive statistics (*M, SD, n*, %) were calculated for the total sample, and separately for participants assigned to the SIT and CAT conditions. Differences in potential confounders were tested with independent sample *t*-tests or χ^2^-tests. To examine group differences over time, repeated measures analyses of variance (rANOVAs) were calculated with the factors time (baseline vs. post-intervention), group (SIT vs. CAT), and time x group interactions. Following Cohen (53), effect sizes were interpreted as follows: η^2^ ≥ 0.01 (small effect), η^2^ ≥ 0.059 (moderate effect), η^2^ ≥ 0.138 (large effect). Between-group differences in meeting physical activity recommendations at baseline and post-intervention were examined via χ^2^-tests. With regard to affective valence, two separate difference scores were built to take baseline levels into account, using the following formula: (a) affective valence during exercise minus affective valence at baseline, (b) affective valence after exercise minus affective valence at baseline. These difference scores were averaged (a) across all sessions, and (b) for every week in order to obtain baseline-controlled indices for affective valence during and after exercise. Subsequently, independent sample *t*-tests (SIT vs. CAT) and rANOVAs were performed separately for affective valence during and after exercise. In the rANOVAs, the factors time (week 1, week 2, week 3, week 4), group (SIT vs. CAT), and time x group were considered.

## Results

### Sample Characteristics and Group Differences in Potential Confounders

Table 1 contains information about the characteristics of the sample, medication at baseline, and baseline differences in potential confounders between the SIT and CAT group. As shown in Table 1, the two groups did not differ with regard to any of the variables. Accordingly, no covariates were taken into account in the subsequent analyses.

**Table 1:** Descriptive statistics and baseline differences.

	**Total sample (*N* = 50)**	**SIT (*n* = 25)**	**CAT (*n* = 25)**		
***Metric variables***	***M* (*SD*)**	***M* (*SD*)**	***M* (*SD*)**	***t***	***d***
Age (years)	36.4 (11.3)	36.4 (12.4)	36.5 (10.4)	−0.05	0.00
Height (cm)	169.1 (9.2)	171.0 (9.5)	167.1 (8.7)	1.51	0.43
Weight (kg)	68.4 (14.7)	67.7 (14.6)	69.0 (15.2)	−0.30	−0.09
BMI (kg/m^2^)	23.8 (4.0)	23.0 (3.5)	24.6 (4.4)	−1.44	0.40
Systolic BP (mmHG)	110.0 (14.5)	109.9 (15.2)	110.1 (13.9)	−0.03	−0.01
Diastolic BP (mmHG)	74.6 (10.1)	74.4 (10.6)	75.0 (9.8)	−0.18	0.06
***Categorical variables***	***n*** **(%)**	***n*** **(%)**	***n*** **(%)**	**χ^2^**	***phi***
Sex				0.12	−0.05
Women	39 (78)	19 (76)	20 (80)		
Men	11 (22)	6 (24)	5 (20)		
Smoking status				0.40	0.09
Smoking	14 (28)	8 (32)	6 (24)		
Non-smoking	36 (72)	17 (68)	19 (76)		
***Medication at baseline***	***n (%)***	***n (%)***	***n (%)***		
**SNRIs**					
Velafaxin (75, 175, 225 mg)	7 (14)	3 (12)	4 (16)		
Mirzapin (7.5, 15, 30 mg)	11 (22)	6 (24)	5 (20)		
Wellbutrin (150 mg)	2 (4)	1 (4)	1 (4)		
**SSRIs**					
Trittico (25, 50, 100 mg)	5 (10)	3 (12)	2 (8)		
Escitalopram (10, 20 mg)	11 (22)	6 (24)	5 (20)		
Citalopram (20 mg)	4 (8)	1 (4)	3 (12)		
Fluoxetin (20, 40 mg)	2 (4)	0 (0)	2 (8)		
Paroxetin (20 mg)	1 (2)	1 (4)	0 (0)		
Cipralex (60 g)	1 (2)	1 (4)	0 (0)		
Seralin (50 mg)	1 (2)	0 (0)	1 (4)		
**Atypical neuroleptic medication**					
Quetiapin (25, 50, 150, 300 mg)	4 (8)	3 (12)	1 (4)		
Trimipramin (50 mg)	1 (2)	0 (0)	1 (4)		

*Data from metric variables expressed as means (M) and standard deviations (SD). Data from categorical variables expressed as frequencies (n) and percentage (%).BMI, body mass index; SIT, sprint interval training; CAT, continuous aerobic exercise training; SNRIs, Serotonin and norepinephrine reuptake inhibitors; SSRIs ,Selective Serotonin Reuptake Inhibitors*.

### Effects of SIT and CAT on Self-Determined Sport and Exercise Motivation

Increases in intrinsic and identified motivation from baseline to post-intervention in the entire study population are visible in Table 2. A significant time effect occurred for the exercise and sport self-determination index, whereas no significant changes were found for introjected and extrinsic motivation.

**Table 2:** Differences between participants assigned to SIT and CAT conditions across time.

					**Unadjusted**					
	**SIT (*****n*** **=** **25)**		**CAT (*****n*** **=** **25)**		**Time**		**Group**		**Time x Group**	
	**Baseline**	**Post**	**Baseline**	**Post**						
	***M* (*SD*)**	***M* (*SD*)**	***M* (*SD*)**	***M* (*SD*)**	***F***	**η*^**2**^***	***F***	**η*^**2**^***	***F***	**η*^**2**^***
**EXERCISE MOTIVATION**										
Intrinsic motivation	2.4 (0.9)	2.6 (0.8)	2.7 (0.7)	2.9 (0.7)	7.7^*^	0.13	1.9	0.04	0.0	0.00
Identified motivation	3.1 (0.8)	3.3 (0.6)	3.1 (0.7)	3.3 (0.6)	5.5^*^	0.10	0.0	0.00	0.2	0.01
Introjected motivation	2.4 (0.7)	2.4 (0.9)	2.4 (0.8)	2.5 (0.7)	0.2	0.00	0.0	0.00	0.4	0.01
Extrinsic motivation	1.5 (0.6)	1.5 (0.5)	1.6 (0.7)	1.5 (0.7)	0.7	0.01	0.0	0.00	0.1	0.00
Self-determination index	2.4 (2.9)	3.1 (2.6)	3.0 (2.5)	3.7 (3.1)	6.9^*^	0.13	0.5	0.01	0.00	0.00
Cardiorespiratory fitness (VO_2_max)	33.1 (8.6)	33.9 (9.1)	31.6 (6.3)	32.9 (6.6)	6.3^*^	0.13	0.3	0.01	0.4	0.01
Perceived fitness	3.6 (2.1)	5.1 (1.9)	4.1 (2.0)	5.4 (1.8)	45.0^***^	0.48	0.5	0.01	0.4	0.01
**SELF-REPORTED PHYSICAL ACTIVITY**										
MPA (min/week)	48 (78)	56 (58)	28 (32)	142 (155)	11.4^**^	0.19	3.0	0.06	8.8^**^	0.16
VPA (min/week)	46 (84)	75 (87)	33 (45)	102 (183)	6.6^*^	0.12	0.1	0.00	1.2	0.02
MVPA (min/week)	94 (141)	130 (123)	60 (65)	244 (290)	12.3^**^	0.20	1.1	0.02	5.60^*^	0.10
Depressive symptoms	31.8 (10.8)	18.8 (12.7)	35.2 (10.3)	23.1 (11.4)	109.8^***^	0.70	1.6	0.03	0.2	0.00

*Data expressed as means (M) and standard deviations (SD). MPA, moderate physical activity; VPA, vigorous physical activity; MVPA, moderate-to-vigorous physical activity*.

* < 0.05;

** p < 0.01;

*** *p < 0.001*.

### Effects of SIT and CAT on Affective Valence

**Figure 2:**
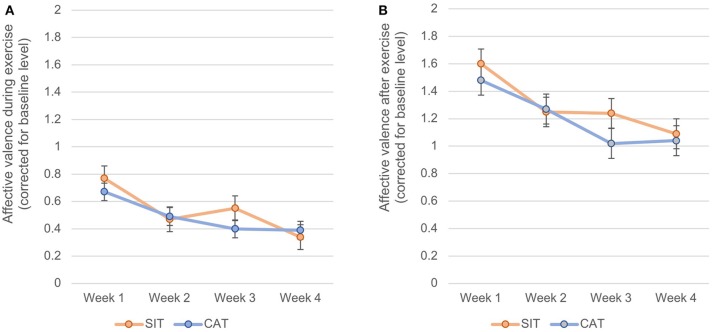
Changes of affective valence during exercise **(A)** and after exercise **(B)** from the first to the last week of intervention, separately for SIT and CAT.

With regard to affective valence *during* exercise, an independent sample *t*-test showed that across all exercise sessions, participants in the SIT (*M* = 0.55, *SD* = 0.48) and CAT (*M* = 0.48, *SD* = 0.64) conditions did not significantly differ from each other, *t*_1, 48_ = 1.35, *p* = 0.25, *d* = −0.12. A rMANOVA yielded a significant decrease in affective valence during exercise from the first to the last week of the intervention, as shown by a significant main effect for time, *F*_3, 46_ = 3.34, *p* < 0.05, χ ^2^ = 0.179. By contrast, no significant effects were found for group, *F*_1, 48_ = 0.08, *p* = 0.774, χ^2^ = 0.002, or time x group, *F*_3, 46_ = 0.65, *p* = 0.590, χ^2^ = 0.040. Figure 2A shows that affective valence was positive in both groups and across all weeks. However, scores decreased from *M* = 1.54 (*SD* = 1.44) in the first week to *M* = 1.04 (*SD* = 1.02) in the fourth week.

A similar pattern of results emerged for affective valence *after* exercise. Again, an independent-sample *t*-test showed that after taking into account baseline affective valence, participants assigned to SIT (*M* = 1.30, *SD* = 0.74) and CAT (*M* = 1.20, *SD* = 1.06) did not differ in their mean scores, *t*_1, 48_ = 1.23, *p* = 0.272, *d* = −0.11. A rMANOVA yielded that affective valence after exercise significantly decreased from the first to the last week of the intervention, as expressed by a significant main effect for time, *F*_3, 46_ = 3.00, *p* < 0.05, χ^2^ = 0.164. Nevertheless, these decreases were similar in the SIT and CAT group, as expressed by an insignificant main effect for group, *F*_1, 48_ = 0.14, *p* = 0.714, χ^2^ = 0.003, and an insignificant time x group interaction effect, *F*_3, 46_ = 0.48, *p* = 0.696, χ^2^ = 0.031. As shown in Figure 2B, affective valence during exercise was positive in both groups and across all intervention weeks. Nevertheless, mean scores dropped from *M* = 0.72 (*SD* = 0.95) in the first week to *M* = 0.37 (SD = 0.66) in the fourth week.

### Effects of SIT and CAT on Fitness, Physical Activity, and Depressive Symptoms

With regard to cardiorespiratory fitness, the significant time effect showed that an improvement occurred in the total sample (see Table 2). Nevertheless, no significant time x group interaction was observed. A similar pattern of results appeared for subjectively perceived fitness.

**Figure 3:**
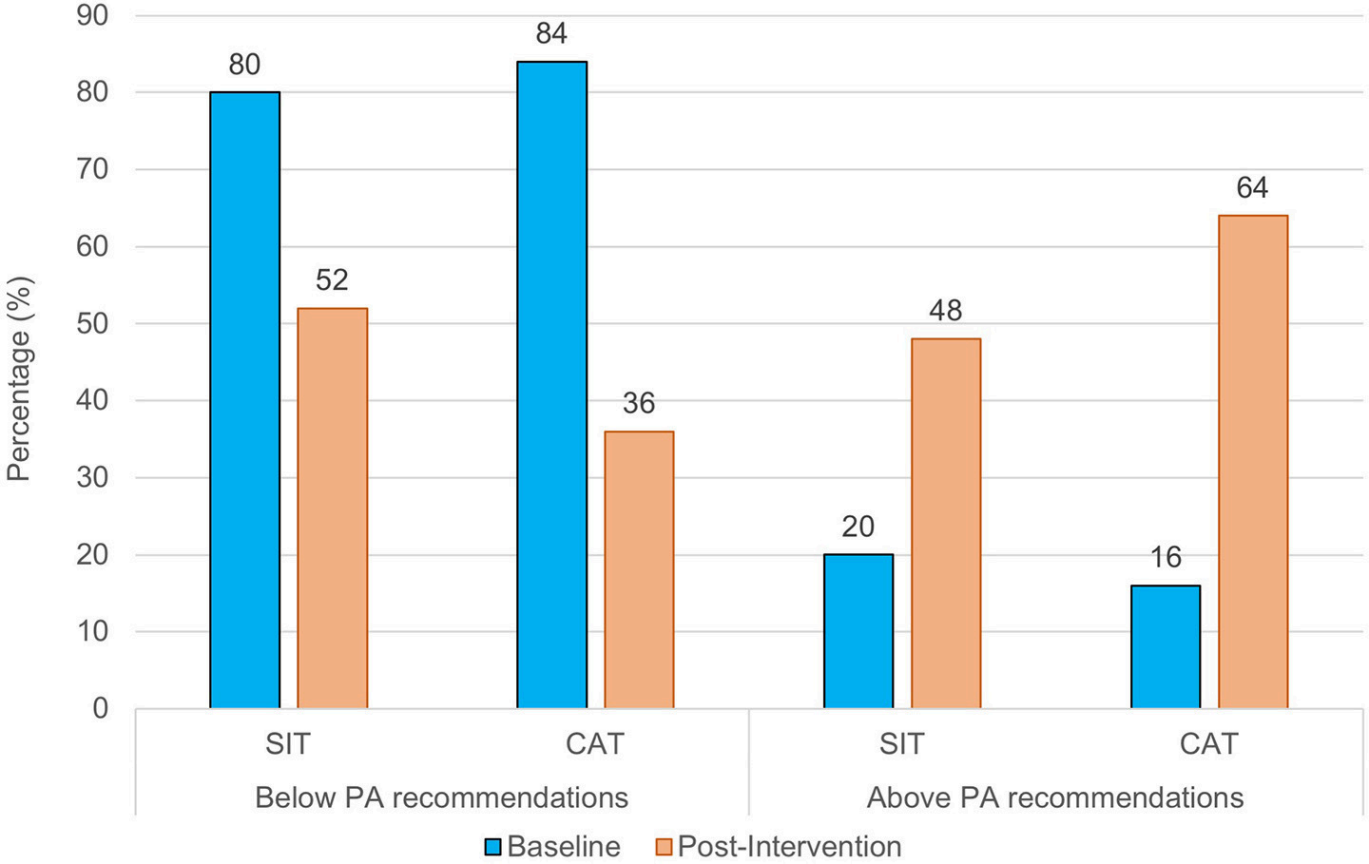
Percentage of participants who meet vs. do not meet physical activity recommendations, separately for SIT and CAT, at baseline and post-intervention.

With regard to self-reported physical activity, significant main effects for time were found for MPA, VPA, and MVPA, indicating that physical activity levels increased in the total sample (see Table 2). Nevertheless, a significant time x group interaction revealed that self-reported levels of MPA and MVPA increased more in the CAT than in the SIT group. As shown in Figure 3, the likelihood of meeting recommended levels of physical activity was slightly higher in the CAT group post-intervention compared to the SIT group. However, both at baseline, $\chi_{1,\;45}^{2}$ = 0.14, *p* = 0.713, *phi* = 0.05, and post-intervention, $\chi_{1,\;45}^{2}$ = 1.30, *p* = 0.254, *phi* = 0.161, group differences were not statistically significant.

As shown in Table 2, a strong reduction in depressive symptoms was observed in both groups, expressed by a significant time effect. However, no significant time x group effect was found, indicating that depressive symptoms similarly decreased in the SIT and CAT group.

## Discussion

Four findings of the present study deserve special attention: First, the SIT and CAT groups did not differ with regard to changes in self-determined sport and exercise motivation from baseline to post-intervention. Second, participants in SIT and CAT showed similar (positive) affective responses *during* and *after* the training sessions. Third, cardiorespiratory fitness, self-perceived fitness, and depressive symptoms similarly improved in the SIT and CAT group. Fourth, significant increases were observed in MPA, VPA, and MVPA from baseline to post-intervention in the total sample. However, these increases were larger in the CAT compared to the SIT group.

As highlighted in the introduction, heated debates are observed in the scientific community regarding the advantages and disadvantages of HIIT in general and SIT in particular (25, 26). Some scholars hypothesized that HIIT/SIT may have a negative impact on participants' motivation, affective valence, adherence, and ultimately future exercise engagement. To date, however, only few studies have examined these assumptions empirically, a shortcoming which was addressed in the current investigation.

Given that criticism has especially focused on the appropriateness of HIIT/SIT in sedentary populations, it is noteworthy that in our study 82% of the participants initially did not meet recommended physical activity standards (<150 min/week of MVPA and <75 min/week of VPA). Moreover, in the present study, we used a Wingate-based interval protocol consisting of 25 repetitions of 30 s of high-intensity intervals at 80% of VO_2_max on a bicycle ergometer, followed by 30 s of complete rest. This deserves special emphasis because Wingate-based protocols are a particularly arduous form of SIT, and are therefore considered especially unsuitable for sedentary populations. Importantly, even proponents of HIIT acknowledge that such a protocol might cause negative affect in untrained individuals or inexperienced exercisers (26). Nevertheless, our findings suggest that from a motivational point of view, the concerns regarding such a training protocol seem unjustified. While intrinsic and identified motivation increased similarly over time in SIT and CAT, introjected, and extrinsic motivation remained stable from baseline to post-intervention. This finding is important because in previous research increased self-determined motivation predicted future physical activity and exercise behavior (28).

Furthermore, affective responses were similar in the SIT and CAT groups. This is at odds with previous studies showing that high-intensity exercise activities cause more negative affect than continuous moderate-intensity exercise, particularly during the exercise session itself (33, 54). However, these findings are difficult to generalize to HIIT or SIT protocols because they were derived from continuous high-intensity exercise protocols. Our findings are also at odds with Ekkekakis et al.'s (55) dual-mode model of exercise-associated affect. This model proposes that differences in affective response during exercise activities of low-to-moderate intensity are primarily attributable to cognitive influences. By contrast, during exercise protocols with higher intensity levels, the importance of interoceptive influences markedly increases. Moreover, the dual-mode model suggests that if exercise intensity levels remain below the ventilatory threshold, affective responses to exercise are positive among most participants. Individual differences increase if intensity levels approach this threshold, and affective responses to exercise become negative for most participants if exercise intensity exceeds the ventilatory threshold. The latter is most likely due to an increased accumulation of lactic acid at higher intensities (cp. 53). We assume that the positive affect observed during SIT in the present study might be attributable to the relatively short sprint intervals. In other words, such short sprint intervals followed by short sequences of complete rest might not lead to an accumulation of lactic acid as observed in continuous high-intensity protocols of longer duration. Alternatively, Weston et al. (14) assumed that for some patients, short bursts of activity may be a more appealing option than the prospect of continuously exercising for an extended period of time at the same intensity. In summary, our study suggests that (baseline-adjusted) affective responses *during* and *after* training are similar (and generally positive) in SIT and CAT. Our findings confirm that affective responses are more positive *after* compared to *during* exercise. Schneider and Kwan (54) argue that focusing exclusively on affective valence *after* exercise may obscure inter-individual differences in affective responding *during* exercise. However, the latter proved to be a particular relevant predictor of physical activity behavior (33, 56).

Our findings also show that similar improvements in depressive symptoms occurred in participants assigned to the SIT and CAT condition. This finding is in line with a previous study showing that exercise therapy is valuable as an add-on to standard care in the treatment of MDD (57). However, due to the absence of a non-exercise control group, it was not possible to judge to what degree the improvements observed in our study were attributable to the exercise intervention.

Our findings further show that cardiorespiratory fitness can be improved to a similar degree via SIT or CAT. Our results are in accordance with a recent meta-analysis by Stubbs et al. (9) showing that clinically relevant improvements in fitness can be achieved through exercise trainings in people with depression. Nevertheless, our findings expand existing research because few studies have attempted to compare SIT and CAT in psychiatric populations. With regard to the magnitude of the improvements, the observed effect size (η^2^ = 0.13) suggests that the impact of the exercise training on cardiorespiratory fitness was moderate to large. Given that the exercise program lasted only 4 weeks, this finding is encouraging and highlights that more systematic efforts are needed to involve psychiatric patients in exercise therapy (58). From a public health perspective, this finding is relevant because psychiatric patients often have lower fitness and physical activity levels compared to healthy controls (59), which is seen as a potential reason for the existing mortality gap between people with and without psychiatric illnesses (60).

Our study further shows that participation in SIT or CAT can lead to strong improvements (η^2^ = 0.48) in self-perceived fitness. The fact that similar improvements occurred in SIT and CAT is noteworthy because scholars have assumed that SIT may lead to feelings of incompetence, failure and decreased self-efficacy (25). Furthermore, improvements in self-perceived fitness are relevant from a therapeutic point of view because high perceived fitness levels were found to be associated with increased psychological well-being in prior investigations (46, 61).

The only significant time x group interactions were observed with regard to self-reported MPA and MVPA. While the SIT and CAT groups reported similar MPA and MVPA levels at baseline, the CAT group reported higher levels post-intervention. This might be an indication that participants in the CAT condition were more willing/motivated to engage in MPA beyond the prescribed exercise training. While this may indicate that CAT has an advantage with regard to physical activity promotion in patients with MDD, this conclusion remains provisional until further evidence is available based on objective accelerometry and longer follow-up periods, covering the time after discharge from the clinic. Increasing levels of MVPA are important from a public health perspective because cardiorespiratory fitness and physical activity proved to be associated independently with lower cardiovascular risk in previous research (62).

Our study suggests that from a motivational point of view SIT seems less problematic than expected. However, this conclusion should be interpreted in light of several limitations. First, we did not assess data regarding the acceptability of SIT and CAT. Our recruitment flow chart shows that among the 195 patients screened for eligibility, 51% were explicitly not willing to participate in the study. This finding was expected given the generally lower motivation of psychiatric patients to engage in physical activity, and the presence of particular exercise barriers in this population (63, 64). However, given the random group assignment, it was not possible to determine whether participation rates would have been higher if participants could have freely chosen between SIT or CAT. Second, we only assessed two specific motivational variables (self-determined motivation and affective valence). We did not assess other constructs such as self-efficacy beliefs, outcome expectations, or enjoyment. Moreover, we assessed depressive symptoms and physical activity levels only via self-reports instead of using clinical interviews or objective accelerometer data, which may have introduced a certain bias (48). Third, we also did not examine how the two intervention conditions influenced patients' automatic evaluations (e.g., implicit attitudes) toward exercise, although recent research shows that such unconscious processes play a role in the regulation of exercise behavior (65). Fourth, our intervention took place in a strongly controlled setting under constant supervision of an exercise physiologist. It therefore remains unclear if patients would have been able to carry out SIT alone without the support of a qualified professional. Fifth, we observed similar dropout rates in the SIT and CAT groups (17–19%). However, longer-term adherence and impact on physical activity behavior after discharge were not assessed. Moreover, because of missing data in the primary outcome (self-determined exercise and sport motivation), the number of included cases dropped below the minimal sample size calculated (50 instead of 52 participants). It is also important to mention that our sample was underpowered to identify effects of small or moderate magnitude. Sixth, we cannot be fully sure whether the observed changes in self-determined sport and exercise motivation (and the other outcomes) are attributable to the two exercise interventions or to “natural” changes during in-patient treatment, as our study design did not include a non-intervention control group or an adjunct sham arm. Seventh, while we presented information about medication of patients at baseline, we did not systematically assess changes in medication during the study period and duration of current depressive episode before treatment. Finally, self-reported MPA increased more strongly from baseline to post-intervention in the CAT group. However, this time x group effect was not mirrored in the CAT group in any of the other study variables.

## Conclusion

This study shows that SIT and CAT have comparable positive effects on self-determined sport and exercise motivation, affective responses, objectively assessed and subjectively perceived fitness, and depressive symptoms among patients with MDD. Thus, from a motivational point of view, SIT seems just as suitable as CAT in the treatment of patients with MDD. This is a promising finding because regarding self-determination theory, it seems advantageous for patients to choose between different exercise therapy regimes. In other words, it might be recommendable to consider patients' preferences with regard to exercise type and intensity. However, this conclusion should be considered provisional until the findings of this study are replicated or corroborated for other HIIT/SIT protocols, and until more knowledge is available regarding how SIT impacts participants' physical activity behavior after they have been discharged from the hospital.

## Availability of Data and Material

Data and material can be requested for further analyses or transparency reasons from the corresponding author.

## Consent for Publication

Consent was obtained from the participants, collaborators, and co-authors.

## Author Contributions

MG, AM, JB, LZ, and LD developed the study design. MG conducted the statistics and wrote the manuscript. All authors contributed to the data interpretation, and the internal revision of the manuscript draft. All authors approved the final draft version.

### Conflict of interest statement

The authors declare that the research was conducted in the absence of any commercial or financial relationships that could be construed as a potential conflict of interest.
